# Cushion Presence and Species Pools Mitigate the Effect of Climate on Species Richness in Alpine Communities

**DOI:** 10.34133/research.0975

**Published:** 2025-10-30

**Authors:** Quansheng Fu, Richard Michalet, Sergei Volis, Xianhan Huang, Juntong Chen, Xinjian Zhang, Qun Liu, Jianwen Zhang, Xiangguang Ma, Jipei Yue, Dacai Zhang, Deli Peng, Yang Niu, Jianguo Chen, Bo Song, Dong Luo, Yang Yang, Pengrui Luo, Xinyuan Kuai, Guo Shi, Zhimin Li, Hang Sun, Tao Deng

**Affiliations:** ^1^ Yunnan Key Laboratory of Plant Diversity and Biogeography, Kunming Institute of Botany, Chinese Academy of Sciences, Kunming 650201, Yunnan, China.; ^2^ Yunnan International Joint Laboratory for Biodiversity of Central Asia, Kunming Institute of Botany, Chinese Academy of Sciences, Kunming 650201, Yunnan, China.; ^3^ University of Chinese Academy of Sciences, Beijing 100049, China.; ^4^ UMR Environnements et Paléoenvironnements Océaniques et Continentaux, University of Bordeaux, Talence FR-33405, France.; ^5^ Faculty of Forestry, Southwest Forestry University, Kunming 650204, Yunnan, China.; ^6^ School of Life Sciences, Yunnan Normal University, Kunming 650500, Yunnan, China.

## Abstract

Abiotic and historical factors are major determinants of large-scale patterns of species richness, yet facilitative interactions can strongly influence diversity in low-productivity habitats such as alpine ecosystems. Cushion plants often promote the establishment of other species, but the relative roles of climate, species pools, and facilitation remain largely unknown. We analyzed 454 plots (4 × 4 m each) of vascular plants from the subnival belts of the Qinghai–Tibet Plateau, using generalized linear mixed models and structural equation modeling. Community species richness was shaped by climate, regional species pools, and cushion presence, with cushions exerting the strongest positive effect in the Hengduan Mountains and Tibetan Plateau. In the structural equation model, cushion presence exerted the strongest positive effect on species richness, whereas climate affected species richness mainly through indirect pathways: wetter conditions enlarged species pools, whereas colder conditions increased cushion presence, which in turn enhanced local species richness. Cushions also buffered the negative effects of aridity. In contrast, species richness variation in the relatively wetter regions of the Himalaya was primarily determined by abiotic factors but not by cushion presence, consistent with the dominant assumption that facilitation is not frequent under favorable climatic conditions. Our findings demonstrate that alpine species richness emerges from the combined effects of species pools and facilitation rather than direct climate effects alone, highlighting the need to integrate biotic and abiotic drivers when explaining biodiversity patterns in extreme environments.

## Introduction

The geographical distribution of species richness (SR) and its driving factors are important topics in biogeography and macroecology [1–3]. Species pools and climate are considered critical determinants of the geographic patterns of SR [4,5]. According to the species pool hypothesis, regional species pools are the outcome of regional and historical processes, whereas local communities are generally regarded as subsets of these pools filtered by local environmental conditions and ecological interactions. Therefore, the size and composition of the regional species pool can leave a lasting imprint on local SR [6–8]. Various climate-related hypotheses have been presented to explain the mechanisms behind the geographic patterns of species diversity, such as the environmental energy hypothesis [9,10], the water–energy dynamics hypothesis [11,12], the freezing tolerance hypothesis [13,14], the climatic seasonality hypothesis [15,16], and the Pleistocene climatic fluctuations hypothesis [17]. These hypotheses mainly address regional or continental diversity patterns and are less applicable to the local (community) scale. Community assembly is primarily shaped by local environmental filtering and biotic interactions, which jointly determine which species from the regional pool can establish and persist under local conditions [2,18]. However, recent studies suggest that SR in extreme environments may be largely decoupled from macroclimate, challenging the prevailing view that climatic factors, particularly those related to water and energy availability, are the primary determinants of the geographic patterns of SR [14,19–22]. One explanation for this pattern is that SR in extreme environments is shaped by the interaction between macroclimatic constraints and other stress factors, with facilitation playing a key role. Facilitation (positive interactions), defined as non-trophic interspecific interactions that increase the average individual fitness of one species (so-called nurse effects) [23–26], has been shown to be an important additional driver of plant communities in many habitats [27–32]. The importance of positive interactions involving nurse plants has been demonstrated in habitats such as deserts, salt marshes, cold alpine and Arctic tundra [25,26,33], and tropical and subtropical habitats [34,35].

A well-known example of nurse plants in alpine ecosystems is the cushion plant [26,30]. Cushion plants, found extensively in high mountain ecosystems globally, are a particular group of nurse plants that have fundamental importance to these ecosystems [36,37]. They act as ecosystem engineers at both the individual and population levels, improving local soil conditions, providing shelter for the seedlings of other species, and enhancing ambient temperature and humidity, thereby promoting the establishment of other species and increasing SR within alpine communities [23,36,38]. Some studies have indicated that the importance of facilitation increases monotonically with environmental severity (i.e., stress), a pattern known as the stress-gradient hypothesis [23,33]. However, an alternative view has emerged, suggesting that facilitation should prevail under intermediate-stress conditions but collapse, or even shift back to competition, under extremely harsh environments [39], thereby producing a unimodal pattern of facilitation along stress gradients [40,41]. This alternative perspective has rapidly gained attention and received empirical support across a variety of natural systems [39,42–45]. These findings indicate that facilitation may contribute disproportionately to species diversity in extreme environments and that its role has likely been either overestimated or underestimated. Cushion plants have been found to positively influence SR in alpine plant communities [29,32,38,46], but their relative contribution and variation, in comparison with abiotic effects and regional species pools, require further investigation.

Importantly, the presence of cushion plants or their co-occurrence with local species does not necessarily constitute evidence of facilitation by cushion plants. Specifically, if the same climatic factors simultaneously drive both the local species pool and the occurrence of cushion plants, the observed co-occurrence at the regional scale may simply reflect a habitat-sharing effect rather than demonstrating a facilitative role of cushion plants in local community diversity [47,48]. For example, at a small scale, facilitators and beneficiary species often share similar microhabitats, as in the “oasis effect” frequently observed in alpine or Arctic ecosystems [47,49]. Neglecting such habitat-sharing effects may statistically bias the results toward overestimation of facilitation [47]. This phenomenon may also arise at a large scale if both community SR and cushion presence are influenced by regional species pools. It is therefore essential to quantify the regional species pool and assess its potential relationships with climate, the presence of cushion plants, and community SR, in order to disentangle facilitation from habitat-sharing effects.

The alpine subnival belt, a truly extreme habitat, is defined as the region with discontinuous vegetation cover above the high mountain vegetation zone and below the snow zone (i.e., the area permanently covered by snow). The climatic extremes include very low temperatures, large diurnal temperature ranges, and high ultraviolet radiation, whose effects are further intensified by patchy vegetation cover and poorly developed scree soils dominated by loose stones and rock fragments [50,51]. In contrast to alpine ecosystems of other regions of the world (e.g., the high Andes and Alps), the vegetation of the alpine subnival belt on the summits of the Qinghai–Tibet Plateau (QTP) has been largely overlooked in earlier alpine studies on alpine vegetation. The flora of the QTP, the highest plateau in the world, is known to be highly adapted to cold environments and particularly vulnerable to warming temperatures due to the limited opportunities for habitat expansion upward [52–54]. Given its top altitudinal position within terrestrial ecosystems [50,51], the alpine subnival belt provides a critical lens for studying plant adaptation and survival under the harshest abiotic stresses in order to disentangle the respective contributions of biotic and abiotic factors shaping the geographic patterns of plant diversity in extreme environments.

Here, we explore the relationships among SR, abiotic variables, species pools, and biotic interactions in the vascular plant communities of the alpine subnival belt on the QTP. We applied a suite of analyses to a plant community dataset comprising 454 plots and 7,242 species records obtained through systematic field surveys. Our specific questions are as follows: (a) What are the relative contributions of cushion plant presence, climate and soil variables, and the regional species pool in explaining the geographic patterns of plant diversity in the alpine subnival zone? (b) Does climate indirectly influence local community SR by simultaneously shaping the regional species pool and the occurrence of cushion plants?

## Results

**Fig. 1.**
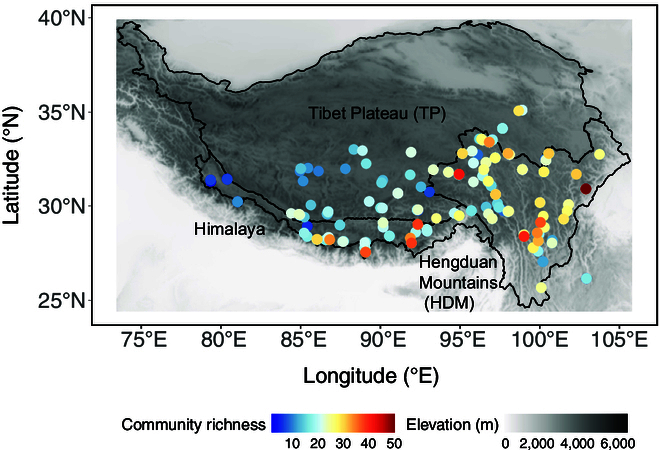
Plant species richness in 454 observation plots of the alpine subnival belt of the Qinghai–Tibet Plateau.

The SR was higher in the eastern and southeastern regions of the QTP than in the western and northwestern regions (Fig. 1). SR was higher in the eastern Hengduan Mountains (HDM) and the southern Himalaya, whereas no clear spatial pattern was observed within the Tibetan Plateau (TP).

**Fig. 2.**
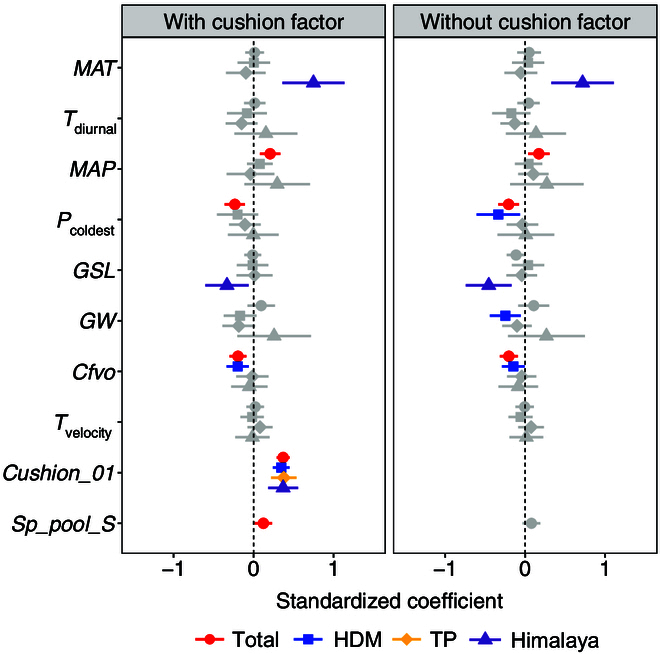
The model-averaged estimates of standardized coefficients (points) and the 95% confidence intervals (bars) derived from the Poisson generalized linear mixed-effects models (GLMMs) (with and without cushion plants as a factor) fit to the species richness. Overall and individual regions are represented by different shapes and colors. Nonsignificant coefficients are in gray. *MAT*, mean annual temperature; *T*_diurnal_, mean diurnal temperature range; *MAP*, mean annual precipitation; *P*_coldest_, precipitation of the coldest quarter; *GSL*, growing season length; *GW*, Gams angle rainfall continentality index; *Cfvo*, coarse fragments volumetric content; *T*_velocity_, temperature velocity; *Cushion_01*, presence (1 for presence and 0 for absence) of cushion plant species within each plot; *Sp_pool_S*, regional species pool; HDM, Hengduan Mountains; TP, Tibetan Plateau.

Abiotic factors played an important role in determining SR (Fig. 2 and Table S1). When only abiotic factors were considered in their effects on SR (estimate ± standard error), there was a negative effect of precipitation of the coldest quarter (*P*_coldest_) (−0.207 ± 0.066, *P* < 0.01) and coarse fragments volumetric content (*Cfvo*) (−0.204 ± 0.057, *P* < 0.001) but a positive effect of mean annual precipitation (*MAP*) (0.171 ± 0.068, *P* < 0.05) on SR for the total area. There were negative effects of *P*_coldest_ (−0.336 ± 0.138, *P* < 0.05), *Cfvo* (−0.148 ± 0.072, *P* < 0.05), and Gams angle rainfall continentality index (*GW*) (−0.248 ± 0.099, *P* < 0.05) on SR for HDM. For the Himalaya, there was a positive effect of mean annual temperature (*MAT*) (0.718 ± 0.199, *P* < 0.001) but a negative effect of growing season length (*GSL*) (−0.456 ± 0.146, *P* < 0.01) on SR. None of the factors had a significant effect for TP. For the total area, abiotic variables explained 10.4% of the total variation in SR (Table S2). For the 3 regions, the variation in SR explained by the abiotic variables ranged from 9.4% (HDM) and 5.7% (TP) to 42.6% (Himalaya) (Table S2).

**Fig. 3.**
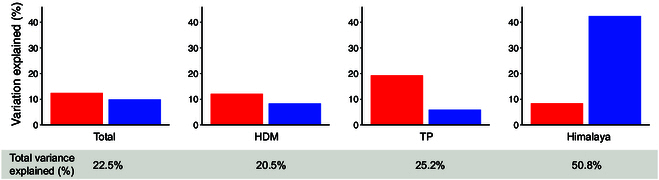
Partitioning of the variance in species richness for the total study area and 3 regions, as explained by biotic variables (i.e., the cushion effect; represented in red) and abiotic variables (including current climate, soil factor, and regional species pool; represented in blue).

In the model that incorporated the cushion factor, both abiotic and biotic drivers contributed substantially to SR (Fig. 2 and Table S3). Among all variables,the cushion factor had the strongest effect among the variables included on SR both for the total area and for 2 of the 3 regions (HDM and TP). There were negative effects of *P*_coldest_ (−0.234 ± 0.065, *P* < 0.001) and *Cfvo* (−0.195 ± 0.054, *P* < 0.001) but positive effects of *MAP* (0.207 ± 0.065, *P* < 0.01), *Cushion_01* (absence/presence of cushion plants) (0.370 ± 0.041, *P* < 0.001), and *Sp_pool_S* (0.123 ± 0.056, *P* < 0.05) on SR for the total area. For HDM, *Cfvo* had a negative effect (−0.198 ± 0.071, *P* < 0.01), but *Cushion_01* had a positive effect on SR (0.346 ± 0.053, *P* < 0.001). For TP, *Cushion_01* (0.378 ± 0.081, *P* < 0.001) had a positive effect on SR. In the Himalaya, *MAT* (0.749 ± 0.198, *P* < 0.001) and *Cushion_01* (0.370 ± 0.096, *P* < 0.001) had positive effects, whereas *GSL* (−0.333 ± 0.138, *P* < 0.05) had a negative effect on SR. All variables explained 22.5% of the total variation in SR for the total area, 20.5% for HDM, 25.2% for TP, and 50.8% for the Himalaya (Fig. 3 and Table S2).

**Fig. 4.**
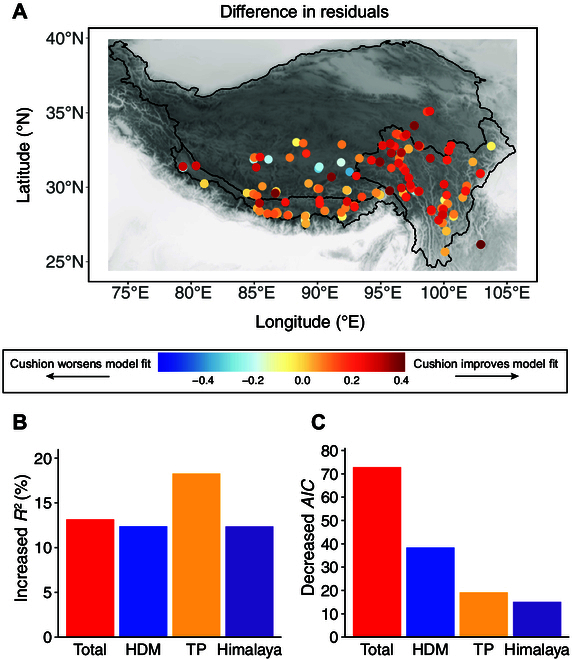
(A) Differences in residuals between the comprehensive model including the biotic factor (cushion presence) and the abiotic-only model. Positive values indicate that residuals in the comprehensive model are closer to zero, reflecting a better model fit. (B) Increases in explained variance (*R*^2^) after including the biotic factor in the model for the total region and each subregion (HDM, TP, and Himalaya). (C) Decreases in Akaike information criterion (*AIC*) after including the biotic factor, indicating improved model performance.

The proportion of variation in SR independently explained by the biotic factor exceeded that explained by abiotic variables, both at the overall regional scale and in 2 out of the 3 subregions examined (Fig. 3 and Table S4). The inclusion of the biotic variable (i.e., cushion presence) significantly enhanced model fitness for predicting SR across all models, resulting in reduced residuals, increased *R*^2^ values, and decreased Akaike information criterion (*AIC*) values (Fig. 4 and Table S2).

**Fig. 5.**
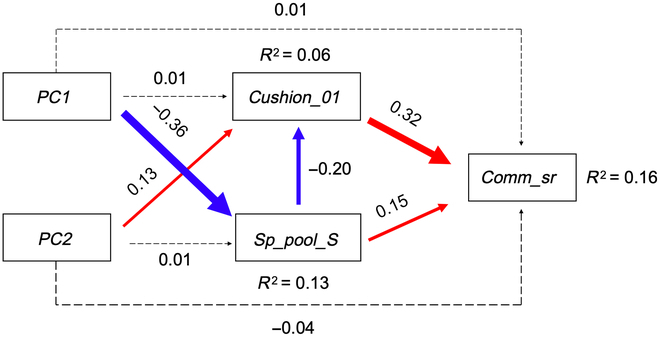
Structural equation model showing the direct and indirect effects of climatic gradients (*PC1*: humidity–continentality; *PC2*: temperature–microhabitat), regional species pool size, and cushion plant presence on community species richness. Solid red arrows indicate significant positive pathways, and solid blue arrows indicate significant negative pathways. Dashed arrows represent nonsignificant relationships (*P* > 0.05) that were retained in the model to meet d-separation requirements. Numbers along the arrows denote standardized path coefficients, and arrow width is proportional to effect size. Coefficients of determination (*R*^2^) for endogenous variables are shown next to the corresponding response boxes. *Cushion_01*, presence (1 for presence and 0 for absence) of cushion plant species within each plot; *Sp_pool_S*, regional species pool; *Comm_sr*, community species richness.

The structural equation model explained 16% of the variation in community SR (Fig. 5). Climate axes (*PC1*: humidity–continentality gradient; *PC2*: temperature–microhabitat gradient) had no significant direct effect on community SR. Instead, we detected 3 significant indirect pathways. First, colder conditions (high *PC2* scores) positively influenced cushion plant presence, which in turn had a strong positive effect on community SR. This indicates that cushion plants buffer the negative effects of cold environments by facilitating the establishment of co-occurring species. Second, wetter conditions (low *PC1* scores) positively affected the size of the regional species pool, which then exerted a positive effect on community SR. This suggests that large-scale climatic wetness enhances local SR primarily through its effect on the availability of species in the regional pool. Third, we found an additional, more complex pathway linking dryness, the species pool, cushion plants, and community SR (*PC1* → *Sp_pool_S* → *Cushion_01* → *Comm_sr*). Drier conditions reduced the size of the species pool, which in turn increased the likelihood of cushion plant presence. Cushion plants subsequently exerted a positive effect on community SR, thereby buffering the negative effect of dryness on local community SR.

## Discussion

Our study demonstrates that SR in the alpine subnival belt of the QTP is shaped by a combination of abiotic conditions, regional species pools, and facilitative interactions, with their relative contributions differing strikingly across regions. At the plateau-wide scale, cushion plant presence was the strongest predictor of local SR, surpassing climatic and edaphic factors. Structural equation modeling further revealed that climate influenced SR mainly through indirect pathways: wetter conditions expanded the regional species pool, and a larger species pool exerted a positive effect on community SR. Colder conditions promoted cushion plant presence, which in turn strongly enhanced community richness. Cushions also mitigated the negative impacts of dryness on community SR by promoting local coexistence when the regional species pool contracted. At the regional scale, cushion effects dominated in HDM and TP, while in the Himalaya—the most benign subregion—abiotic factors explained a larger share of variation, likely because wetter conditions expanded the species pool and competition prevailed under favorable environments. Taken together, these findings highlight that community assembly in the alpine subnival belt is not governed by direct climatic control, but rather arises from the interactive effects of climate, regional species pools, and facilitation.

Importantly, our results indicate that the effects of environmental gradients on local SR were not primarily direct, but were mediated through 2 distinct pathways: (a) the humidity–continentality gradient indirectly influenced SR by positively affecting the size of the regional species pool, and (b) the temperature–microhabitat gradient indirectly influenced SR by promoting the presence of cushion plants. Among these pathways, cushion presence exerted the strongest positive effect on local SR, whereas the contribution of the species pool was comparatively weaker. These findings demonstrate that alpine community SR is simultaneously shaped by facilitation and species pool effects. This supports the idea that facilitation can enhance SR in nonsaturated communities by recruiting species from the regional pool under intermediate environmental severity [55]. We also found that the presence of cushion plants reduced the negative effects of drought and low temperatures on local community SR, suggesting that cushions may act as a buffering or insurance mechanism that alleviates the decline in SR under increasingly severe environmental conditions, consistent with previous studies [31,38,56]. Moreover, our results show that the positive direct effect of cushions on local SR is unlikely to reflect habitat-sharing processes at the regional scale, since species pool size and cushion presence were driven by different mechanisms in the structural equation model. Facilitation in alpine environments has been shown to result mainly from environmental severity effect rather than neighborhood effects [56]. This implies that cushions do not necessarily improve the performance of species growing beneath them, but rather reduce the negative impacts of harsh conditions on species lacking nurse plants, thereby limiting the negative effect of environmental severity on SR.

The univariate analyses provide additional mechanistic insights into the structural equation model results. Among the abiotic variables used in the analyses for the total region, only mean annual precipitation (*MAP*) and regional species pool (*Sp_pool_S*) showed positive effects on SR, whereas precipitation in the coldest quarter (*P*_coldest_) and coarse fragments volumetric content (*Cfvo*) had negative effects. The positive effect of *MAP* is consistent with the idea that higher water availability supports the presence of more species [2,11,57]. This finding also supports the results reported by Ma et al. [58] and Cheng et al. [59], who demonstrated a positive relationship between *MAP* and community SR in alpine grasslands and deserts of QTP. We also observed a negative impact of high *Cfvo* on SR. In the soils of the alpine subnival belt, rocks and coarse fragments usually dominate. An increased rock fraction reduces the soil’s capacity to retain water and organic matter, thereby decreasing plant biomass and vegetative cover [60–62]. Loose and small stones forming scree, the predominant topographic type in the subnival zone of the QTP, make it challenging for roots to proliferate and establish optimally [63,64] because the gravel on the slope can easily move downward, shifting the aboveground parts of plants away from their subterranean root systems [65]. A typical example is *Rheum nobile*, in which we observed that the displacement distance between the aboveground parts and the upslope-located root systems can reach up to 2 m (Tao Deng, personal observations). These univariate effects are consistent with the structural equation model framework: *MAP* corresponds to the “wet → species pool expansion → SR increase” pathway, and *Cfvo* reflects negative effects of edaphic stress, which can be partly buffered by cushion plants at the local scale.

Nevertheless, all abiotic variables explained only a small proportion of the variance in SR (17.0% on average across the total region and 3 subregions). This may be attributed to 3 possible reasons. Firstly, species inhabiting high-altitude and subarctic zones could colonize the alpine subnival belt because of their preadaptation to such extreme environments. The ancestors of many seed plant clades originated during the warm interval between the early Cretaceous and the late Eocene [66,67]. However, with global cooling, plant clades adapted to warmer climates in high-latitude regions faced a critical choice: migrate to more temperate latitudes, develop cold tolerance, or go extinct [68]. Due to phylogenetic niche conservatism, only a small number of clade members are capable of crossing major ecophysiological boundaries, particularly into harsher environments such as colder ones [69,70]. However, some plants inhabiting the alpine subnival belt have developed specialized morphological structures and physiological adaptations that allow them to survive winter temperatures below freezing, without crossing major ecophysiological boundaries [71–74]. For instance, “greenhouse plants”, e.g., *Saussurea obvallata* and *R. nobile*, have leaves or translucent bracts that act as a greenhouse, increasing the temperature within the flowers, while “woolly plants”, e.g., *Saussurea medusa*, have dense bracts with bushy hairs covering the inflorescence, helping to maintain an optimal temperature. Some subnival plant species accumulate more fat, soluble sugars, and flavonoids than alpine meadow plants to resist frost [75]. Secondly, many of the species occupying the alpine subnival belt of the QTP may be descendants of the ancestral clades with similar ecological niches. The distribution patterns of plants in cold environments may be driven by similar formative processes and mechanisms [76]. Recent studies have proposed that as the difference between current and ancestral ecological niches increases, the explanatory power (i.e., *R*^2^) of contemporary climate in explaining patterns of SR increases [77]. The uplift of the QTP during 3.6 to 1.5 Ma and the subsequent climate changes during the mid-Pleistocene (1.17 Ma) have driven rapid speciation and intraspecific differentiation within many taxa in this plant community [78,79]. Compared to species originating in other regions and subsequently migrating into the QTP, many native species exhibit minimal differences between their current and ancestral ecological niches, which may weaken the explanatory power of contemporary climate. Thirdly, the lack of a temperature effect on SR may be attributed to the relatively small variation in temperature conditions across the QTP [59] and particularly among the studied locations of the subnival zone (annual temperature range from −8.2 to +5.6 °C). Jiménez-Alfaro et al. [20], who also found a neutral effect of temperature on SR in the alpine grasslands of Central and Southern Europe, attributed this lack of an effect to “filtering” of their study system (alpine grasslands) by low-temperature conditions. These findings provide additional support for the weak direct effects of climate detected in the structural equation model.

A key finding of our study is that, even at a relatively fine regional scale, the nursing effect of the cushion plants explains variation in species diversity within subnival belt vegetation better than climatic and soil variables. This implies that cushion plants play a primary driving role in the plant communities under extreme environmental conditions. Cushion plants are known to be essential for maintaining and promoting plant diversity in high mountain regions [80–83], as they can improve local soil conditions, provide shelter, and enhance the germination, growth, and establishment of other species. However, the relative strength of the positive interactions provided by cushion plants in determining the community composition, and therefore its SR, in comparison with the major abiotic factors has remained largely unexplored. A positive effect of cushion plants on alpine community SR was clearly shown in a number of studies [32,38,80], but the only study comparing the strength of abiotic and biotic (nursing) effects by Cavieres et al. [38] found that the effect sizes of climatic variables on total SR at the global scale were higher than those of the facilitator cushion species. There are 2 reasons for the inconsistency in the results of our studies. First, Cavieres et al. did not use the presence of cushion plants or the number of cushion plants in each plot as an independent variable, but instead used “the proportion of increase in non-cushion species richness”. Second, their study system had less extreme environmental conditions than our study system (1.5 °C vs. −4.7 °C mean annual temperature and 835 mm vs. 557 mm mean annual precipitation). This further supports our finding that in the alpine subnival belt—one of the harshest environments worldwide—the relative contribution of facilitation can be greatly amplified.

As a regional contrast, another interesting finding of our study is that the variability in the explanatory power of variables for species diversity patterns at the regional scale was higher than that at the larger scale (i.e., 3 regions were combined). For instance, the variation in SR explained by both biotic and abiotic variables for the Himalaya was 50.8%, while for TP and HDM, it was 25.2% and 20.5%, respectively. This indicates that the relationship between species diversity and the environmental conditions varies from one region to another, and the magnitude of these differences is such that it can make a relationship estimated at a higher geographical scale statistically weak or misleading.

Notably, in the Himalaya, the variance explained by cushion presence (8.4%) was far lower than that explained by abiotic factors (42.4%). Regional comparisons revealed that Himalayan plots occupied the most benign environments among the 3 regions (high water availability and low diurnal temperature range) and spanned the widest climatic gradients. According to the theory that competition may dominate under favorable environmental conditions [23,41,84], competitive interactions are likely to prevail in the Himalaya, thereby reducing the potential for facilitation by cushions [85]. Additionally, consistent with the structural equation model results, the wetter climate in the Himalaya enhanced the size of the regional species pool, suggesting that the species pool pathway was the dominant driver of local SR in this region. Furthermore, the broader climatic gradient in the Himalaya relative to those in HDM and TP may have strengthened the explanatory power of abiotic factors. Taken together, these effects likely explain why abiotic gradients accounted for more variation in SR than cushion presence in the Himalaya.

Although our study is based on a large dataset of plots covering a large area, there are still some limitations. The first limitation arises from the mismatch in geographical scale between vegetation plots (only 4 × 4 m) and the coarse-grained environmental predictor variables that we utilized (ca. 1 km^2^), as emphasized in other biogeographical analyses [5,21,38]. However, we addressed this issue by conducting resampling procedures equivalent to climate resolution, which demonstrated the robustness of our results. Future work with microclimate and fine-scale edaphic layers could refine these estimates further. A second limitation is that, although many studies have suggested a facilitative role of cushion species on species growing within cushions, the extent of their effects on species beyond their immediate vicinity remains to be investigated [80]. Additionally, interactions between plants and other organisms not included in our analysis, such as pollinators, seed dispersers, specialized herbivores, and soil microorganisms, may also influence species survival and diversity patterns in extreme environments [86–88]. Indeed, the relatively low percentage of variation in SR explained by both environmental factors and cushion species suggests that dispersal-related processes, microclimatic conditions, inherent stochasticity in species occurrences, and other spatially structured effects have important impacts on local SR patterns [89].

Nevertheless, our work provides the first systematic quantification of the relative contributions of abiotic factors, regional species pools, and facilitation in the alpine subnival belt, one of the most extreme terrestrial environments on Earth. The findings not only underscore the foundational role of cushion plants as key facilitators but also highlight that the combined effects of cushion plants and regional species pools can buffer the negative impacts of harsh climatic stresses such as drought and low temperatures on local community diversity, thereby offering new evidence to explain biodiversity patterns under severe conditions. These insights are particularly relevant in the context of rapid climate change and the ongoing biodiversity crisis, and they provide an important scientific basis for developing more effective conservation strategies [90,91].

## Materials and Methods

### Study area

The QTP, with a mean elevation exceeding 4,000 m [92,93], is one of the most biodiverse regions globally, supporting more than 12,000 native vascular plant species [94,95] and around 1,500 species in the alpine subnival belt [96]. Furthermore, the QTP encompasses 2 global biodiversity hotspots, namely, the Himalaya and mountains of southwest China [97–99]. This study focuses on the subnival belt where the plant communities, with highly patchy and fragmented plant cover, usually occur on or between rocks and screes [50,51]. According to WorldClim [100], in the subnival belt of the QTP, the mean annual temperature (based on 454 sampled plots) is −1.8 °C, and the mean temperature of the coldest quarter is −10.4 °C. The mean annual precipitation is 557.2 mm, most of which falls in summer, with 305.8 mm occurring during the warmest quarter. The QTP is traditionally subdivided into 3 primary regions: HDM, the Himalaya, and TP (Fig. 1). Located at the southeastern periphery of the QTP, HDM is characterized by a rugged terrain, featuring north–south-oriented mountain ranges interspersed with deep valleys. Stretching along the southern margin of the QTP, the Himalaya is a narrow, elongated mountain range, where substantial climatic variation occurs due to the rain shadow effect, giving rise to warm and moist southern slopes and cold, arid northern slopes. TP, occupying the vast central and northwestern portions of the QTP, has an average elevation of approximately 4,500 m and is distinguished by a harsh, cold, and arid climate. To characterize regional climatic differences, we compared the 3 study regions along the first 2 principal component analysis axes (Fig. S1; details are provided below) and tested pairwise contrasts using Tukey’s honest significant difference (Fig. S2). *PC1*, representing a humidity–continentality gradient (higher scores = drier and more continental conditions), differed significantly among all 3 regions (*P* < 0.001). TP exhibited the highest *PC1* values, indicating the driest and most continental climate; the Himalaya showed the lowest values, corresponding to the most benign conditions (higher water availability and lower diurnal temperature range); and HDM occupied an intermediate position. In contrast, *PC2*, which reflects a temperature–microhabitat gradient (higher scores = colder conditions, shorter growing seasons, and skeletal soils), did not differ significantly among regions. Overall, strong regional climatic contrasts were captured primarily along *PC1*, whereas variation in *PC2* occurred mainly within regions rather than between them.

### Community data

Plant community data were collected over 2 consecutive years, 2018 and 2019, during the period from July to August when most plants were flowering and thus could be identified to the species level. Data were collected for each of the 454 plots (4 × 4 m in size) established across the QTP. Specifically, there were 265 plots in HDM, 86 in the Himalaya, and 103 in TP. Three plots were located outside HDM, but since they were situated on a mountain peak over 4,000 m, we included them in HDM. In each plot, we recorded every plant species present. For identification, we engaged botanical experts from relevant taxonomic groups and botanical institutions. All voucher specimens were deposited at the Herbarium of the Kunming Institute of Botany. The established 454 plots span a range of altitudes from 3,690 to 6,041 m. We standardized the botanical nomenclature of species using the package U.Taxonstand [101] according to the World Checklist of Vascular Plants [102].

### Abiotic and biotic variables

The climatic variables comprised mean annual temperature (*MAT*), mean diurnal temperature range (*T*_diurnal_), mean annual precipitation (*MAP*), precipitation of the coldest quarter (*P*_coldest_), minimum temperature of the coldest month (*T*_min_), and growing season length (*GSL*, defined as the number of months with mean maximum temperature >12.5 °C). To account for strong rain shadow effects in mountain areas, we calculated the Gams angle rainfall continentality index (*GW*) based on winter precipitation [103]. Summer water balance (*S*_wb_) was computed as the ratio of summer precipitation to the mean of maximum summer temperatures. *MAT*, *MAP*, *T*_min_, *GSL*, *GW*, and *S*_wb_ were downloaded or calculated from the Climate Downscaling Tool [104] at a resolution of 30 arcsec. Mean diurnal range (*T*_diurnal_) and precipitation of the coldest quarter (*P*_coldest_) were downloaded from the WorldClim database [100] (www.worldclim.org) at a resolution of 30 arcsec. In addition, we included temperature velocity (*T*_velocity_), reflecting the rate of climate change since the Last Glacial Maximum [17].

The soil variable was coarse fragments volumetric content (*Cfvo*), which was downloaded from the SoilGrids database [105] at a resolution of 250 m and at a 15-cm depth. To retain the high resolution of the data as much as possible, we did not resample the high-resolution (250-m) soil data to the resolution of the climatic factors (approximately 1 km at the equator).

To account for the potential influence of the regional species pool on local community SR, we estimated gamma diversity for each of the 3 study regions (HDM, TP, and the Himalaya). To ensure comparability across regions with different sampling efforts, we standardized the sample size by randomly selecting 86 plots (equal to the number of plots in the Himalaya, the least sampled region) from each region and calculated the total number of unique species within those plots. The resulting gamma diversity values were then assigned to all plots within the corresponding region, thereby providing a region-level predictor variable (e.g., all plots in HDM were assigned the HDM gamma value, and those in TP, the TP value). Because some taxa, particularly grasses, could not always be identified to the species level, we applied 2 alternative treatments to test the robustness of our estimates. In the strict version (*Sp_pool_S*), unidentified taxa within the same genus were collapsed into a single species (e.g., all unidentified *Poa* assigned to *Poa sp1*). In the relaxed version (*Sp_pool_L*), unidentified taxa within the same genus were treated as potentially distinct species (e.g., *Poa sp1* and *Poa sp30*). Both versions of the species pool variable were incorporated into subsequent models. Results were qualitatively consistent between the 2 treatments, indicating that our conclusions were robust to the treatment of unidentified species.

We chose the presence of cushion plant species within a plot as a potential proxy of biotic interactions because cushion plant species create favorable conditions for the establishment and growth of other species, as extensively demonstrated by previous studies in other regions [26] and our own prior research in the QTP [80–82,106–111]. Following Zhang et al. [112], cushion-forming plant species were classified based on a global database of cushion plant species [113] (http://www.cushionplants.eu/). For each plot, we recorded the presence or absence of cushion species and coded it as a binary variable (*Cushion_01*), with 1 indicating presence and 0 indicating absence.

### Statistical analyses

We first divided our analysis into 2 groups: (a) evaluating the effect of abiotic factors alone on the geographic patterns of species diversity and (b) assessing the combined effects of both biotic and abiotic factors on these spatial diversity patterns.

We fitted Poisson generalized linear mixed-effects models (GLMMs) to assess the relative contributions of environmental variables to SR using the “glmer” function of the lme4 package [114] in R 4.0.5 [115]. In addition, we accounted for spatial autocorrelation by adding a random intercept for 0.05° (approximately 5 km) cells. To control for overdispersion, we incorporated an observation-level random effect by assigning a unique identifier to each plot. We examined pairwise Pearson’s correlation among all variables. To mitigate potential multicollinearity, we retained only those variables that met the criteria of a Pearson’s correlation coefficient (|*r*|) ≤ 0.6 (Fig. S3). The final set of variables incorporated into the model included 10 variables, namely, *MAT*, *T*_diurnal_, *MAP*, *P*_coldest_, *GSL*, *GW*, *Cfvo*, *T*_velocity_, *Sp_pool_S*, and cushion. All continuous variables were standardized by subtracting the mean and dividing by 2 standard deviations, which allowed direct comparisons of the estimated coefficients between continuous variables and untransformed categorical moderator [116]. We conducted model selection by constructing GLMMs for all possible combinations of predictor variables. Model averaging was then applied to subsets of models (delta *AIC* < 4), based on *AIC* weights, to obtain averaged parameter estimates using “model.avg” function of the MuMIn package [117]. We calculated Moran’s *I* values for all model residuals to check spatial autocorrelation in the “testSpatialAutocorrelation” function and checked the presence of overdispersion of fitted models in the “testDispersion” function of the DHARMa package [118]. Marginal and conditional *R*^2^ values (*mR*^2^ and *cR*^2^) were calculated using the “r.squaredGLMM” function of the MuMIn package [117]. Finally, we performed hierarchical partitioning analyses to assess the relative importance of each fixed-effect predictor using the “glmm.hp” function of the glmm.hp package [119,120]. This method allocates equally the shared variance attributable to collinear explanatory variables and has been widely used in recent studies [121–123]. To assess whether the biotic variable has a stronger effect on species diversity than abiotic variables, we partitioned the explained individual *R*^2^ for each variable into 2 groups, one explained independently by biotic variable (cushion) and the other explained independently by abiotic variables, by summing the individual marginal *R*^2^ values of each set of fixed factors.

Finally, we evaluated whether the inclusion of the biotic factor improved the fit of the comprehensive model relative to that of the abiotic-only model, based on 3 criteria: smaller residuals (i.e., closer to zero), higher marginal *R*^2^ (*mR*^2^), and lower *AIC* values. We compared the residual values for each plot between the comprehensive model (including both abiotic and biotic variables) and the abiotic-only model. If the residual of a plot in the comprehensive model was closer to zero compared to that in the abiotic-only model, then the biotic factor improved the model fit for that plot. The difference in residuals was computed by subtracting the absolute residual value of the comprehensive model from that of the abiotic-only model for each plot. We also compared the overall *R*^2^ and *AIC* values between the comprehensive model and the abiotic-only model. If *R*^2^ was higher and the *AIC* value was lower in the comprehensive model compared to those in the abiotic-only model, then the biotic factor improved the model fit for that model. The *R*^2^ and *AIC* values were extracted from the model outcomes.

In addition to performing the overall analysis described above, we also conducted separate analyses for each of the 3 regions, i.e., TP, HDM, and the Himalaya. For each region, the retained variables exhibited certain collinearity, yet all models had variance inflation factor (*VIF*) < 10. We ran models for each region, both excluding collinear factors and without considering collinearity. The estimated coefficients for the 2 sets of models were very similar. This indicates that the models at the regional level are not sensitive to the intercorrelation of environmental variables. Therefore, we present the results using all factors in the main text.

We then used structural equation modeling to disentangle the direct and indirect effects of environmental gradients on community SR. We first performed a principal component analysis on 10 environmental variables to reduce dimensionality and identify dominant gradients structuring alpine plant communities. The variables included *MAT*, *T*_diurnal_, *MAP*, *P*_coldest_, *T*_min_, *S*_wb_, *GSL*, *GW*, *Cfvo*, and *T*_velocity_. All variables were standardized prior to analysis. The first 2 principal components together explained 56.6% of the total variance (*PC1*: 31.3%; *PC2*: 25.3%; Fig. S1). *PC1* loaded positively on *GW* and *T*_diurnal_ but negatively on *MAP*, *S*_wb_, *MAT*, and *P*_coldest_, representing a gradient from, humid environments with high water availability to dry, continental sites with large diurnal temperature ranges (Fig. S1). *PC2* was negatively associated with *MAT*, *T*_min_, and *GSL* but positively with *Cfvo*, capturing a stress axis from warmer sites with long growing seasons to colder, high-stress environments characterized by short growing seasons and skeletal soils (Fig. S1). These 2 axes (moisture and temperature) were retained as composite variables of environmental severity in subsequent models. Structural equation modeling was restricted to the complete dataset. The structural equation model comprised 3 linked submodels: (a) cushion plant presence (*Cushion_01*) modeled with logistic regression using moisture, temperature, and species pool size (*Sp_pool_S*) as variables; (b) species pool size modeled with linear regression using moisture and temperature as variables; and (c) community SR modeled with negative binomial regression to account for overdispersion, with moisture, temperature, species pool size, and cushion presence as variables. Models were fitted using the R package piecewiseSEM [124], and standardized path coefficients were reported.

### Sensitivity analyses

To address potential instability arising from the mismatch between the coarse resolution of climate data and the finer scale of vegetation plots, we randomly selected one plot per 1-km^2^ grid cell (approximately 0.01°, matching the climate data resolution) to construct a subset dataset. Each subset, corresponding to one sampling iteration, contained 208 plots (i.e., one plot per 1-km^2^ cell). We fitted GLMMs to each subset and repeated this resampling procedure 999 times to obtain mean standardized regression coefficients and *R*^2^ using the “model.avg” function of the MuMIn package [117].

To evaluate the sensitivity of the estimation of biotic effect to metric choice, we replicated the analysis using cushion SR within plots (*Cushion_num*) as the biotic predictor.

Comparison of the results of the data subset analysis with the full dataset analysis revealed a very similar coefficient for the cushion, indicating that our models were not sensitive to the coarse resolution of climate data (Figs. S4 and S5 and Table S5) and metrics for quantifying biotic effects (Fig. S6 and Table S6).

## Data Availability

All data needed to replicate these analyses are available in Figshare at https://doi.org/10.6084/m9.figshare.27694809.
