# Supplementary material for: Cushion Presence and Species Pools Mitigate the Effect of Climate on Species Richness in Alpine Communities
Source: Research (Wash D C). 2025 Oct 30;8:0975. doi: 10.34133/research.0975 (PMC12573088; doi:10.34133/research.0975)
Supplement: Supplementary 1 — Figs. S1 to S6 Tables S1 to S6 [file research.0975.f1.docx]

**Supporting information for**

Cushion presence and species pools mitigate the effect of climate on species richness in alpine communities

This PDF file includes:

Figures S1–S6

Tables S1–S6


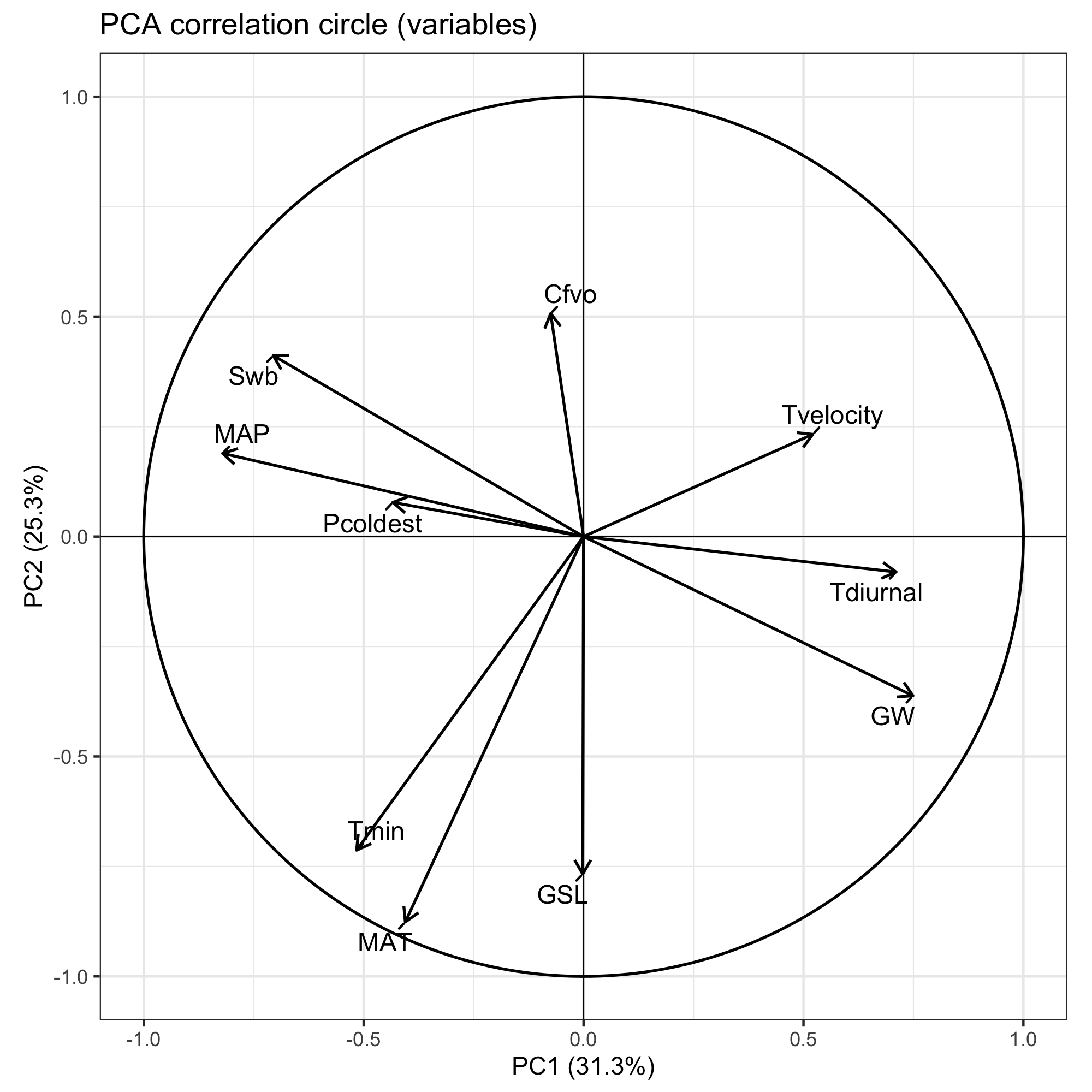


**Fig. S1.** Principal component analysis (PCA) correlation circle showing the loadings of environmental variables on the first two principal components (PC1 and PC2, explaining 31.3% and 25.3% of the total variance, respectively). Abbreviations: mean annual temperature (MAT); mean diurnal temperature range (Tdiurnal); mean annual precipitation (MAP); precipitation of the coldest quarter (Pcoldest); growing season length (GSL); Gams–angle rainfall continentality index (GW); coarse fragments volumetric (Cfvo); temperature velocity (Tvelocity); minimum temperature of the coldest month (Tmin); Summer water balance (Swb).


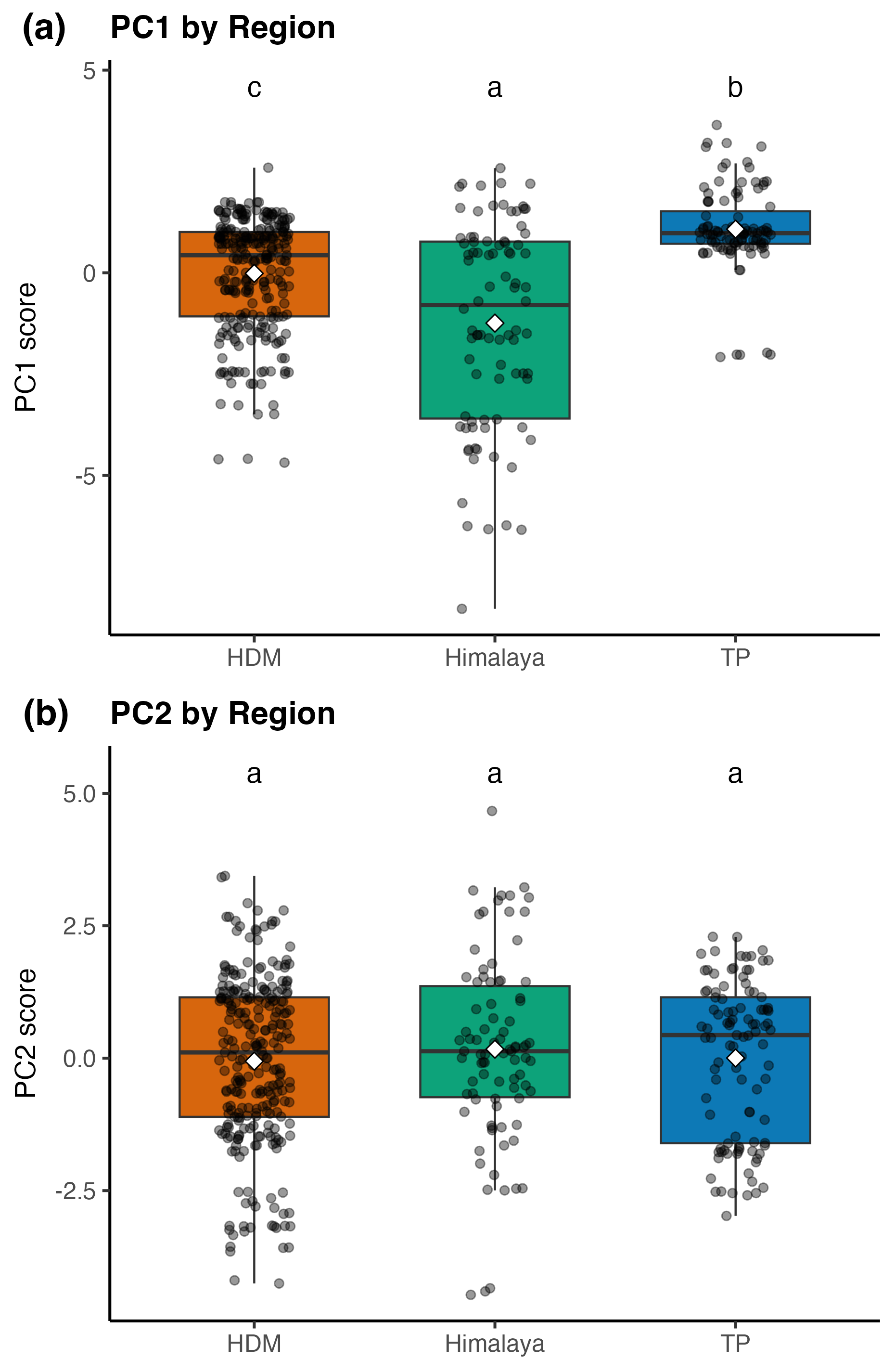


Fig. S2. Regional variation in climatic conditions. (a) PC1, representing a humidity–continentality gradient (higher scores indicate drier and more continental climates), differed significantly among the three regions based on Tukey’s HSD tests (p < 0.001). (b) PC2 showed no significant differences among regions. Letters above boxplots denote significant differences between regions at p = 0.05.


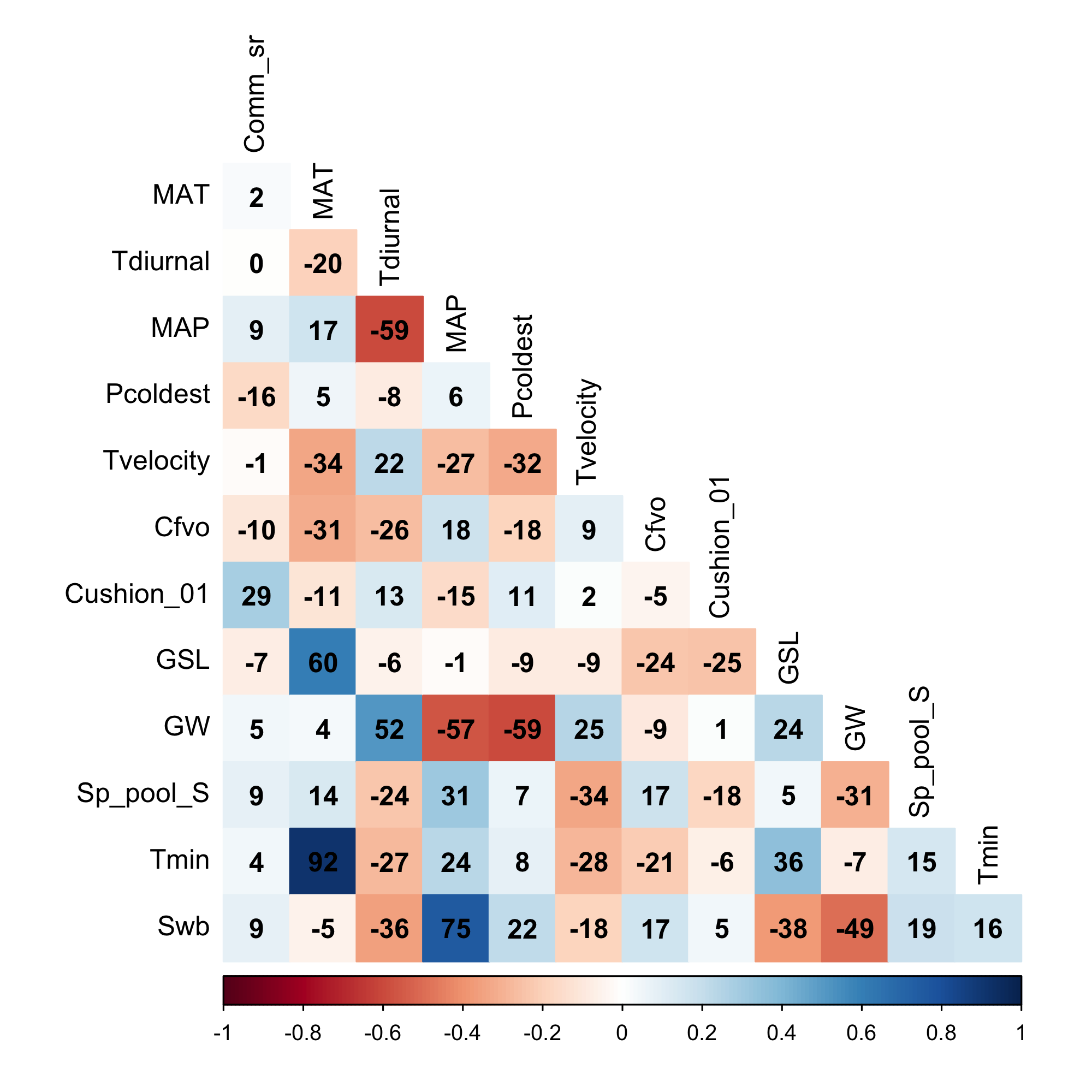
Fig. S3. The Pearson’s correlation coefficients among environmental variables for total area species richness.Abbreviations: mean annual temperature (MAT); mean diurnal temperature range (T_diurnal_); mean annual precipitation (MAP); precipitation of the coldest quarter (P_coldest_); growing season length (GSL); Gams–angle rainfall continentality index (GW); coarse fragments volumetric (Cfvo); temperature velocity (T_velocity_); the presence (1 for presence, 0 for absence) of cushion plant species within each plot (Cushion_01); regional species pool (Sp_pool_S); minimum temperature of the coldest month (T_min_); Summer water balance (S_wb_).


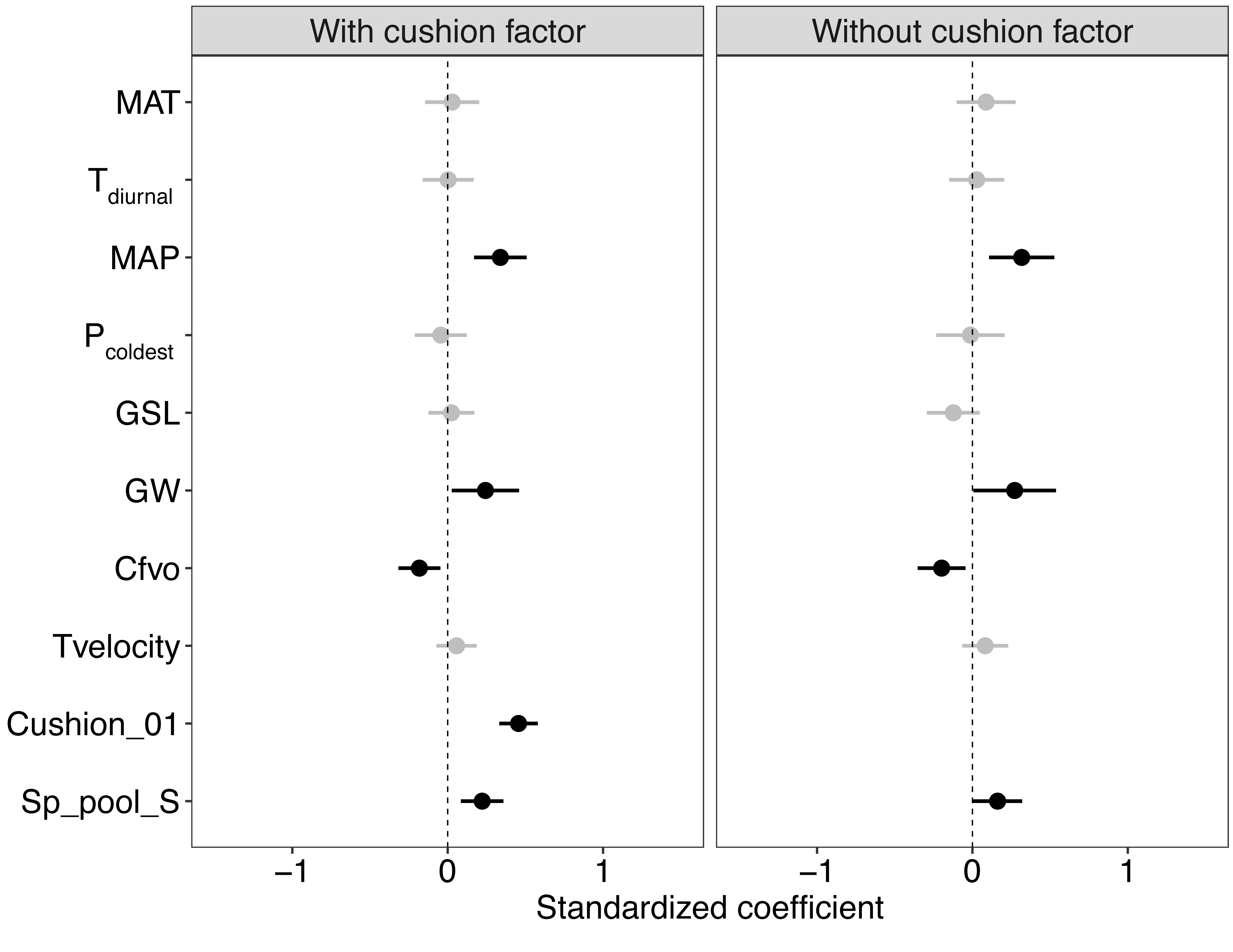


Fig. S4**.** Model results are derived from a weighted average of 999 models fit to 999 random subsets (1km) of all vegetation plots. The model-averaged estimates of standardized coefficients (points) and the 95% confidence intervals (bars) derived from the Poisson generalized linear mixed-effects models (GLMMs) (with and without cushion plants as a factor) fit to the species richness. Nonsignificant variables are shown in gray. Abbreviations: mean annual temperature (MAT); mean diurnal temperature range (T_diurnal_); mean annual precipitation (MAP); precipitation of the coldest quarter (P_coldest_); growing season length (GSL); Gams–angle rainfall continentality index (GW); coarse fragments volumetric (Cfvo); temperature velocity (T_velocity_); the presence (1 for presence, 0 for absence) of cushion plant species within each plot (Cushion_01); regional species pool (Sp_pool_S).


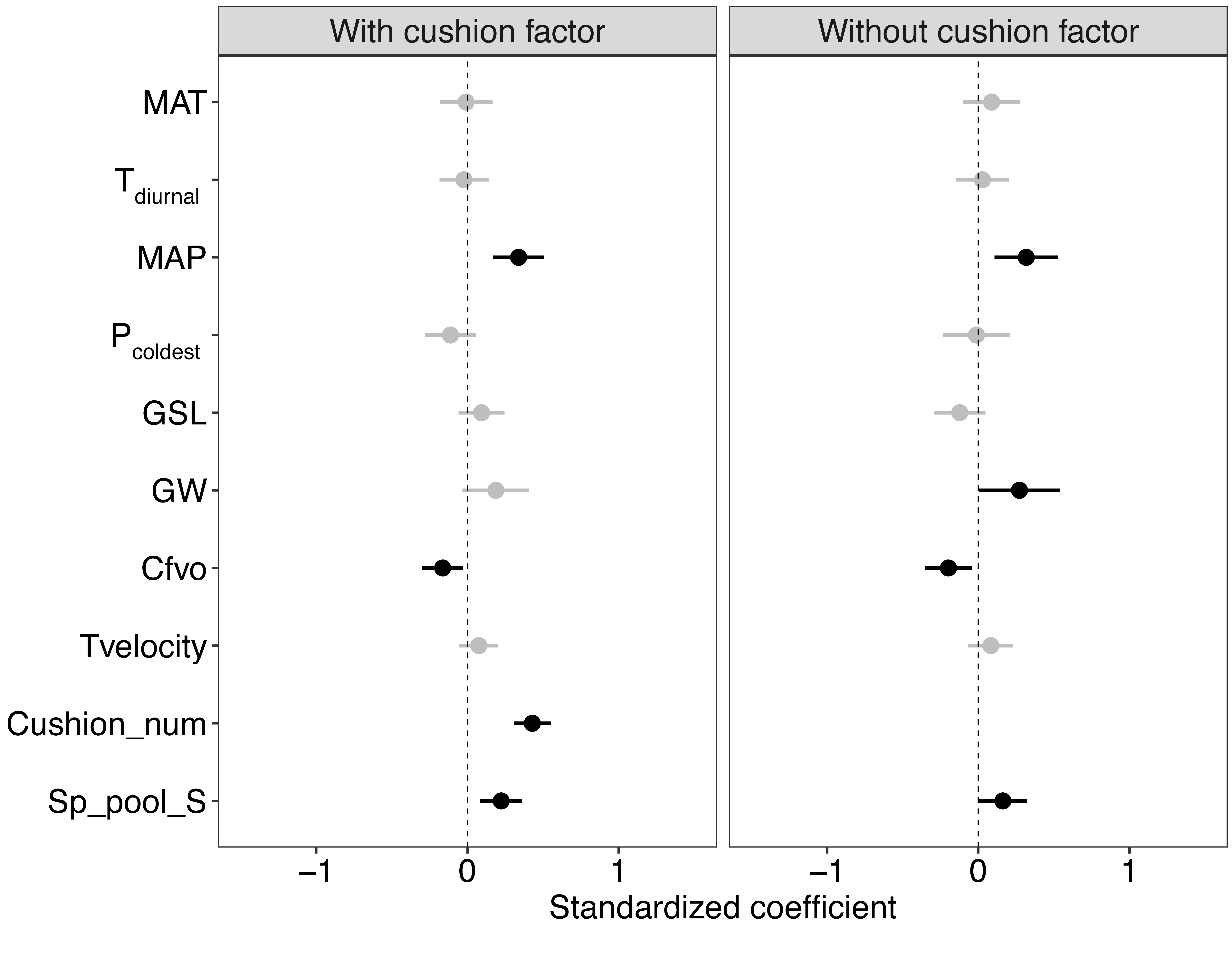
Fig. S5. Model results are derived from a weighted average of 999 models fit to 999 random subsets (1km) of all vegetation plots. The model-averaged estimates of standardized coefficients (points) and the 95% confidence intervals (bars) derived from the Poisson generalized linear mixed-effects models (GLMMs) (with and without cushion plants as a factor) fit to the species richness. Nonsignificant variables are shown in gray. Abbreviations: mean annual temperature (MAT); mean diurnal temperature range (T_diurnal_); mean annual precipitation (MAP); precipitation of the coldest quarter (P_coldest_); growing season length (GSL); Gams–angle rainfall continentality index (GW); coarse fragments volumetric (Cfvo); temperature velocity (T_velocity_); cushion species richness within plots (cushion_num); regional species pool (Sp_pool_S).


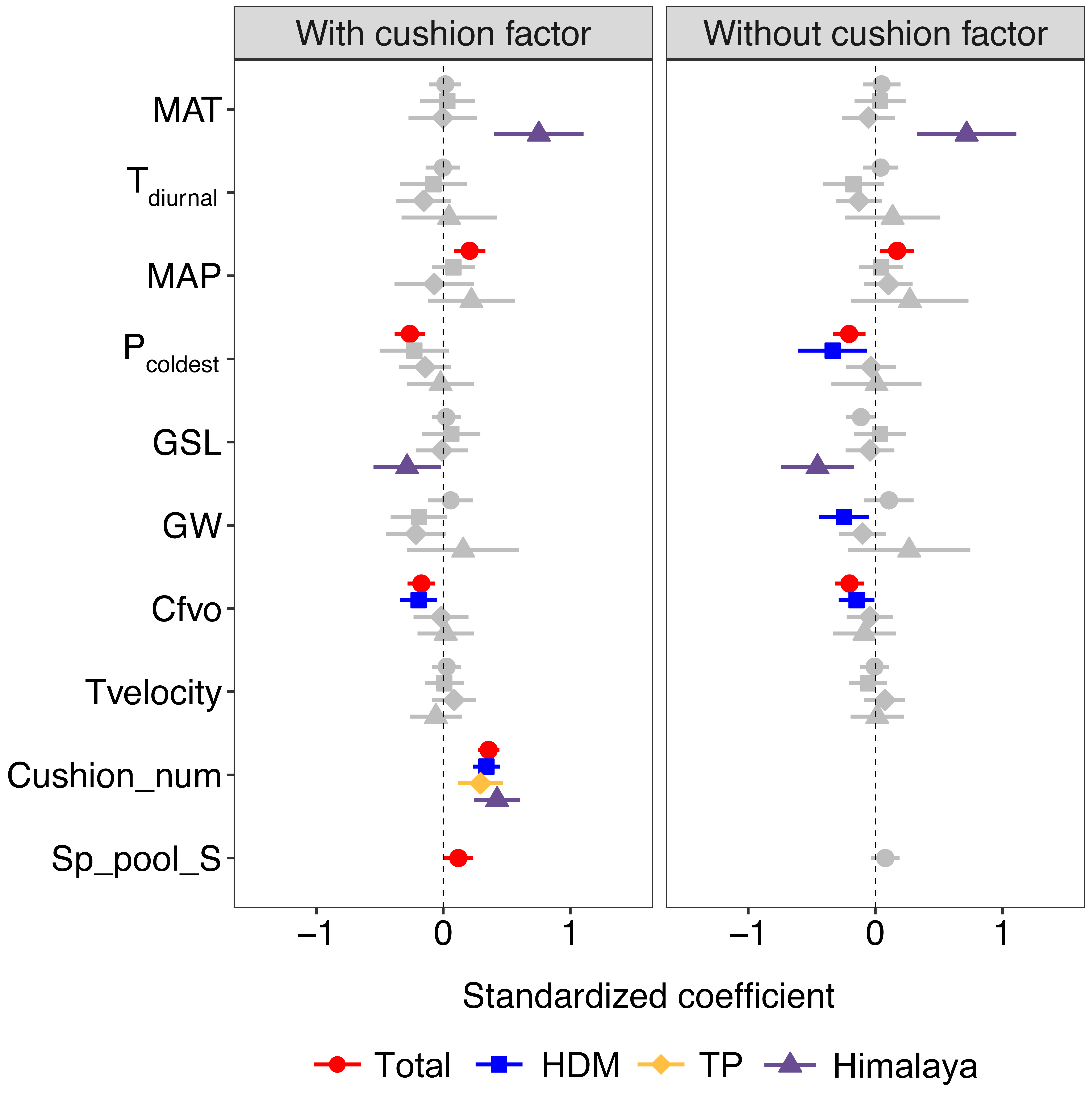


Fig. S6. The model-averaged estimates of standardized coefficients (points) and the 95% confidence intervals (bars) derived from the Poisson generalized linear mixed-effects models (GLMMs) (with and without cushion plants as a factor) fit to the species richness. Overall and individual regions were represented by different shapes and colors. Nonsignificant variables are shown in gray. Abbreviations: mean annual temperature (MAT); mean diurnal temperature range (T_diurnal_); mean annual precipitation (MAP); precipitation of the coldest quarter (P_coldest_); growing season length (GSL); Gams–angle rainfall continentality index (GW); coarse fragments volumetric (Cfvo); temperature velocity (T_velocity_); cushion species richness within plots (cushion_num); regional species pool (Sp_pool_S).

Table S1. Model results for the effects of abiotic factors on patterns of species richness. For the multivariate models, the scaled coefficients, z-values, and p-values of the fixed effects are reported. Models for each region exclude collinear factors. Models for each region (HDM, TP and **Himalaya**) were run without considering collinearity.

| **Without cushion factor** | | | | |
| --- | --- | --- | --- | --- |
| **Variable** | **Estimate** | **Std. Error** | **z value** | **p value** |
| **Total area** | | | | |
| MAT | 0.049 | 0.076 | 0.646 | 0.519 |
| T_diurnal_ | 0.041 | 0.071 | 0.581 | 0.561 |
| MAP | 0.171 | 0.068 | 2.496 | 0.013 |
| P_coldest_ | -0.207 | 0.066 | 3.141 | 0.002 |
| GSL | -0.114 | 0.059 | 1.922 | 0.055 |
| GW | 0.107 | 0.099 | 1.083 | 0.279 |
| Cfvo | -0.204 | 0.057 | 3.562 | 0.000 |
| T_velocity_ | -0.006 | 0.058 | 0.111 | 0.912 |
| Sp_pool_S | 0.078 | 0.057 | 1.366 | 0.172 |
| **HDM** | | | | |
| MAT | 0.037 | 0.103 | 0.358 | 0.720 |
| T_diurnal_ | -0.173 | 0.122 | 1.406 | 0.160 |
| MAP | 0.043 | 0.087 | 0.493 | 0.622 |
| P_coldest_ | -0.336 | 0.138 | 2.428 | 0.015 |
| GSL | 0.036 | 0.103 | 0.349 | 0.727 |
| GW | -0.248 | 0.099 | 2.498 | 0.012 |
| Cfvo | -0.148 | 0.072 | 2.053 | 0.040 |
| T_velocity_ | -0.058 | 0.077 | 0.754 | 0.451 |
| **TP** | | | | |
| MAT | -0.054 | 0.105 | 0.510 | 0.610 |
| T_diurnal_ | -0.130 | 0.092 | 1.399 | 0.162 |
| MAP | 0.103 | 0.097 | 1.050 | 0.294 |
| P_coldest_ | -0.035 | 0.101 | 0.341 | 0.733 |
| GSL | -0.043 | 0.098 | 0.432 | 0.665 |
| GW | -0.102 | 0.094 | 1.065 | 0.287 |
| Cfvo | -0.043 | 0.093 | 0.460 | 0.646 |
| T_velocity_ | 0.074 | 0.082 | 0.894 | 0.371 |
| **Himalaya** | | | | |
| MAT | 0.718 | 0.199 | 3.558 | 0.000 |
| T_diurnal_ | 0.135 | 0.192 | 0.698 | 0.485 |
| MAP | 0.271 | 0.235 | 1.142 | 0.253 |
| P_coldest_ | 0.008 | 0.181 | 0.046 | 0.963 |
| GSL | -0.456 | 0.146 | 3.078 | 0.002 |
| GW | 0.266 | 0.245 | 1.073 | 0.283 |
| Cfvo | -0.086 | 0.127 | 0.671 | 0.502 |
| T_velocity_ | 0.015 | 0.107 | 0.136 | 0.892 |

Table S2. Comparing two metrics for quantifying biotic effects, adding buffer factors improved the model's fit with species richness compared to a model that only considers abiotic factors.

| **Region** | **R^2^** of with cushion model | **R^2^** of without cushion model | **Increased R^2^** (%) | **AIC of with cushion model** | **AIC of without cushion model** | **Decreased AIC** |
| --- | --- | --- | --- | --- | --- | --- |
| **Cushion_01** | | | | | | |
| Total | 0.225 | 0.104 | 0.121 | 2973 | 3043.3 | 70.3 |
| HDM | 0.205 | 0.094 | 0.111 | 1759.8 | 1795.3 | 33.5 |
| TP | 0.252 | 0.057 | 0.195 | 653.6 | 673.4 | 19.8 |
| Himalaya | 0.508 | 0.426 | 0.084 | 569 | 581.9 | 12.9 |
| **Cushion_num** | | | | | | |
| Total | 0.213 | 0.063 | 0.150 | 2983.1 | 3043.3 | 60.2 |
| HDM | 0.206 | 0.067 | 0.139 | 1760.8 | 1795.3 | 34.5 |
| TP | 0.181 | 0.091 | 0.090 | 661.8 | 673.4 | 11.6 |
| Himalaya | 0.531 | 0.348 | 0.183 | 564.5 | 581.9 | 17.4 |

Table S3. Model results for the effects of biotic and abiotic factors on patterns of species richness. Cushion factor was that the presence of cushion plants in the community. For the multivariate models, the scaled coefficients, z-values, and p-values of the fixed effects are reported. Models for each region (HDM, TP and **Himalaya**) were run without considering collinearity.

| **With Cushion_01** | | | | |
| --- | --- | --- | --- | --- |
| **Variable** | **Estimate** | **Std. Error** | **z value** | **p value** |
| **Total area** | | | | |
| MAT | 0.013 | 0.060 | 0.209 | 0.835 |
| T_diurnal_ | 0.014 | 0.068 | 0.209 | 0.834 |
| MAP | 0.207 | 0.065 | 3.168 | 0.002 |
| P_coldest_ | -0.234 | 0.065 | 3.618 | 0.000 |
| GSL | -0.011 | 0.054 | 0.198 | 0.843 |
| GW | 0.095 | 0.088 | 1.084 | 0.278 |
| Cfvo | -0.195 | 0.054 | 3.635 | 0.000 |
| T_velocity_ | 0.020 | 0.056 | 0.360 | 0.719 |
| Cushion_01 | 0.370 | 0.041 | 8.965 | 0.000 |
| Sp_pool_S | 0.123 | 0.056 | 2.203 | 0.028 |
| **HDM** | | | | |
| MAT | 0.005 | 0.104 | 0.044 | 0.965 |
| T_diurnal_ | -0.083 | 0.127 | 0.647 | 0.517 |
| MAP | 0.078 | 0.082 | 0.950 | 0.342 |
| P_coldest_ | -0.200 | 0.132 | 1.514 | 0.130 |
| GSL | -0.012 | 0.101 | 0.123 | 0.902 |
| GW | -0.169 | 0.105 | 1.608 | 0.108 |
| Cfvo | -0.198 | 0.071 | 2.788 | 0.005 |
| T_velocity_ | -0.019 | 0.075 | 0.247 | 0.805 |
| Cushion_01 | 0.346 | 0.053 | 6.478 | 0.000 |
| **TP** | | | | |
| MAT | -0.097 | 0.126 | 0.758 | 0.448 |
| T_diurnal_ | -0.150 | 0.101 | 1.463 | 0.143 |
| MAP | -0.039 | 0.152 | 0.251 | 0.802 |
| P_coldest_ | -0.108 | 0.101 | 1.055 | 0.292 |
| GSL | 0.012 | 0.115 | 0.103 | 0.918 |
| GW | -0.185 | 0.104 | 1.754 | 0.079 |
| Cfvo | -0.016 | 0.103 | 0.150 | 0.881 |
| T_velocity_ | 0.081 | 0.080 | 1.004 | 0.315 |
| Cushion_01 | 0.378 | 0.081 | 4.622 | 0.000 |
| **Himalaya** | | | | |
| MAT | 0.749 | 0.198 | 3.750 | 0.000 |
| T_diurnal_ | 0.154 | 0.201 | 0.761 | 0.447 |
| MAP | 0.296 | 0.209 | 1.402 | 0.161 |
| P_coldest_ | -0.003 | 0.162 | 0.019 | 0.985 |
| GSL | -0.333 | 0.138 | 2.376 | 0.017 |
| GW | 0.257 | 0.235 | 1.081 | 0.280 |
| Cfvo | -0.053 | 0.116 | 0.447 | 0.655 |
| T_velocity_ | -0.014 | 0.110 | 0.127 | 0.899 |
| Cushion_01 | 0.370 | 0.096 | 3.814 | 0.000 |

Table S4. Model results for the partitioning of variance in species richness were obtained using two metrics to quantify biotic effects.

| **Region** | **R^2^_cushion** | **R^2^_climate** | **R^2^_total** |
| --- | --- | --- | --- |
| **cushion_01** | | | |
| Total | 0.125 | 0.099 | 0.225 |
| HDM | 0.121 | 0.084 | 0.205 |
| TP | 0.193 | 0.059 | 0.252 |
| Himalaya | 0.084 | 0.424 | 0.508 |
| **cushion_num** | | | |
| Total | 0.113 | 0.100 | 0.213 |
| HDM | 0.118 | 0.088 | 0.206 |
| TP | 0.105 | 0.076 | 0.181 |
| Himalaya | 0.116 | 0.415 | 0.531 |

| **Without cushion factor** | | | | |
| --- | --- | --- | --- | --- |
| **Fixed effects** | **Estimate** | **Std. Error** | **z value** | **p value** |
| MAT | 0.088 | 0.097 | 0.902 | 0.367 |
| T_diurnal_ | 0.027 | 0.090 | 0.301 | 0.763 |
| MAP | 0.317 | 0.107 | 2.945 | 0.003 |
| P_coldest_ | -0.013 | 0.112 | 0.114 | 0.909 |
| GSL | -0.123 | 0.087 | 1.413 | 0.158 |
| GW | 0.272 | 0.136 | 1.988 | 0.047 |
| Cfvo | -0.198 | 0.079 | 2.511 | 0.012 |
| T_velocity_ | 0.082 | 0.076 | 1.079 | 0.281 |
| Sp_pool_S | 0.162 | 0.081 | 1.994 | 0.046 |
| **With cushion01** | | | | |
| MAT | 0.029 | 0.089 | 0.327 | 0.744 |
| T_diurnal_ | 0.003 | 0.084 | 0.035 | 0.972 |
| MAP | 0.339 | 0.086 | 3.904 | 0 |
| P_coldest_ | -0.044 | 0.085 | 0.517 | 0.605 |
| GSL | 0.024 | 0.076 | 0.318 | 0.75 |
| GW | 0.243 | 0.111 | 2.18 | 0.029 |
| Cfvo | -0.182 | 0.069 | 2.628 | 0.009 |
| T_velocity_ | 0.057 | 0.066 | 0.865 | 0.387 |
| Cushion_01 | 0.456 | 0.063 | 7.173 | 0 |
| Sp_pool_S | 0.222 | 0.070 | 3.161 | 0.002 |
| **With Cushion_num** | | | | |
| MAT | -0.009 | 0.089 | 0.096 | 0.924 |
| T_diurnal_ | -0.023 | 0.083 | 0.282 | 0.778 |
| MAP | 0.338 | 0.085 | 3.937 | 0 |
| P_coldest_ | -0.113 | 0.086 | 1.296 | 0.195 |
| GSL | 0.093 | 0.078 | 1.191 | 0.234 |
| GW | 0.188 | 0.113 | 1.658 | 0.097 |
| Cfvo | -0.164 | 0.068 | 2.372 | 0.018 |
| T_velocity_ | 0.075 | 0.066 | 1.143 | 0.253 |
| Cushion_num | 0.429 | 0.062 | 6.897 | 0 |
| Sp_pool_S | 0.223 | 0.071 | 3.122 | 0.002 |

**Table S5. Model results for the effects of biotic and abiotic factors on species richness patterns across the total area were derived from a weighted average of 999 models, each fit to a random subset (1 km) of all vegetation plots. The cushion factor represents the presence or number of cushion plants in the community. For the multivariate models, the scaled coefficients, z-values, and p-values of the fixed effects are reported.**

Table S6. Model results for the effects of biotic and abiotic factors on patterns of species richness. C**ushion factor was that the number of cushion plants in the community.** For the multivariate models, the scaled coefficients, z-values, and p-values of the fixed effects are reported. Models for each region (HDM, TP and **Himalaya**) were run without considering collinearity.

| **With Cushion_num** | | | | |
| --- | --- | --- | --- | --- |
| **Variable** | **Estimate** | **Std. Error** | **z value** | **p value** |
| **Total area** | | | | |
| MAT | 0.015 | 0.063 | 0.235 | 0.814 |
| T_diurnal_ | -0.004 | 0.068 | 0.058 | 0.954 |
| MAP | 0.207 | 0.062 | 3.312 | 0.001 |
| P_coldest_ | -0.264 | 0.060 | 4.375 | 0.000 |
| GSL | 0.023 | 0.057 | 0.396 | 0.692 |
| GW | 0.057 | 0.089 | 0.641 | 0.522 |
| Cfvo | -0.174 | 0.054 | 3.176 | 0.001 |
| T_velocity_ | 0.026 | 0.057 | 0.456 | 0.648 |
| Cushion_num | 0.357 | 0.042 | 8.372 | 0.000 |
| Sp_pool_S | 0.118 | 0.056 | 2.099 | 0.036 |
| **HDM** | | | | |
| MAT | 0.031 | 0.109 | 0.282 | 0.778 |
| T_diurnal_ | -0.078 | 0.132 | 0.589 | 0.556 |
| MAP | 0.079 | 0.085 | 0.932 | 0.351 |
| P_coldest_ | -0.228 | 0.137 | 1.656 | 0.098 |
| GSL | 0.063 | 0.115 | 0.543 | 0.587 |
| GW | -0.192 | 0.112 | 1.709 | 0.088 |
| Cfvo | -0.194 | 0.073 | 2.651 | 0.008 |
| T_velocity_ | 0.007 | 0.077 | 0.092 | 0.926 |
| Cushion_num | 0.339 | 0.053 | 6.354 | 0.000 |
| **TP** | | | | |
| MAT | -0.004 | 0.136 | 0.029 | 0.977 |
| T_diurnal_ | -0.156 | 0.108 | 1.431 | 0.152 |
| MAP | -0.071 | 0.157 | 0.447 | 0.655 |
| P_coldest_ | -0.143 | 0.102 | 1.387 | 0.166 |
| GSL | -0.011 | 0.102 | 0.108 | 0.914 |
| GW | -0.216 | 0.117 | 1.822 | 0.068 |
| Cfvo | -0.019 | 0.109 | 0.170 | 0.865 |
| T_velocity_ | 0.085 | 0.086 | 0.977 | 0.328 |
| Cushion_num | 0.293 | 0.089 | 3.251 | 0.001 |
| **Himalaya** | | | | |
| MAT | 0.752 | 0.177 | 4.207 | 0.000 |
| T_diurnal_ | 0.046 | 0.188 | 0.240 | 0.810 |
| MAP | 0.221 | 0.170 | 1.281 | 0.200 |
| P_coldest_ | -0.022 | 0.133 | 0.165 | 0.869 |
| GSL | -0.286 | 0.133 | 2.119 | 0.034 |
| GW | 0.156 | 0.222 | 0.695 | 0.487 |
| Cfvo | 0.019 | 0.111 | 0.164 | 0.870 |
| T_velocity_ | -0.059 | 0.104 | 0.559 | 0.576 |
| Cushion_num | 0.423 | 0.090 | 4.618 | 0.000 |
